# Survey of pretreatment HIV drug resistance and the genetic transmission networks among HIV-positive individuals in southwestern China, 2014–2020

**DOI:** 10.1186/s12879-021-06847-5

**Published:** 2021-11-12

**Authors:** Xiaoshan Xu, Liuhong Luo, Chang Song, Jianjun Li, Huanhuan Chen, Qiuying Zhu, Guanghua Lan, Shujia Liang, Zhiyong Shen, Zhiqiang Cao, Yi Feng, Lingjie Liao, Hui Xing, Yiming Shao, Yuhua Ruan

**Affiliations:** 1 National Center for AIDS/STD Control and Prevention, Chinese Center for Disease Control and Prevention, Beijing, 102206 China; 2 Guangxi Key Laboratory of Major Infectious Disease Prevention Control and Biosafety Emergency Response, Guangxi Center for Disease Control and Prevention, Nanning, 530028 China

**Keywords:** HIV, Pretreatment drug resistance (PDR), Antiretroviral therapy (ART), Drug resistance mutations (DRMs), Genetic transmission networks

## Abstract

**Background:**

Pretreatment drug resistance (PDR) can limit the effectiveness of HIV antiretroviral therapy (ART). The aim of this study was to assess the prevalence of PDR among HIV-positive individuals that initiated antiretroviral therapy in 2014–2020 in southwestern China.

**Methods:**

Consecutive cross-sectional surveys were conducted in Qinzhou, Guangxi. We obtained blood samples from individuals who were newly diagnosed with HIV in 2014–2020. PDR and genetic networks analyses were performed by HIV-1 pol sequences using the Stanford HIV-database algorithm and HIV-TRACE, respectively. Univariate and multivariate logistic regression models were used to explore the potential factors associated with PDR.

**Results:**

In total, 3236 eligible HIV-positive individuals were included. The overall prevalence of PDR was 6.0% (194/3236). The PDR frequency to NNRTI (3.3%) was much higher than that of NRTI (1.7%, p < 0.001) and PI (1.2%, p < 0.001). A multivariate logistic regression analysis revealed that PDR was significantly higher among individuals aged 18–29 (adjusted odds ratio (aOR): 1.79, 95% CI 1.28–2.50) or 30–49 (aOR: 2.82, 95% CI 1.73–4.82), and harboring CRF08_BC (aOR: 3.23, 95% CI 1.58–6.59). A total of 1429 (43.8%) sequences were linked forming transmission clusters ranging in size from 2 to 119 individuals. Twenty-two individuals in 10 clusters had the same drug resistant mutations (DRMs), mostly to NNRTIs (50%, 5/10).

**Conclusions:**

The overall prevalence of PDR was medium, numerous cases of the same DRMs among genetically linked individuals in networks further illustrated the importance of surveillance studies for mitigating PDR.

**Supplementary Information:**

The online version contains supplementary material available at 10.1186/s12879-021-06847-5.

## Background

Since the establishment of China’s national free HIV antiviral therapy in 2003, the case fatality rate of HIV infection has been effectively reduced and the life span of people living with HIV/AIDS has been prolonged [1, 2]. However, with the increasing use of antiretroviral drugs, drug resistance has become an urgent problem. Pretreatment drug resistance (PDR) means that drug resistance has been identified by testing the resistance prior to antiviral therapy, including transmitted drug resistance (TDR) or primary resistance, or having received prior treatment, and then restarting antiviral therapy [3]. Studies found that the risk of virological failure within 12 months of treatment in people who developed PDR is 2–3 times higher, which will reduce the long-term effectiveness of first-line therapy [4]. Therefore, timely monitoring of DRMs and the transmission of drug-resistant strains before initiation of ART can provide a scientific basis for large-scale prevention and control program in China.

To address the challenges posed by drug-resistant viruses, WHO formulated a global strategy for the prevention of HIV drug resistance in 2012 [5]. Subsequently, it formulated the monitoring of drug resistant people who initiated antiretroviral therapy [3], which was recommended to monitor HIV resistance levels and factors associated with HIV drug resistance. National surveillance data showed that the prevalence of PDR was still at a low level in China [6–8], but the latest studies showed that PDR has reached a medium level in many parts of the country, such as Shanghai (17.4%, 55/317) [9], Tianjin (11.5%, 35/305) [10], Guangxi (7.21%, 83/1151) [11], and Beijing (6.12%, 57/932) [12]. According to the consolidated guidelines on the use of antiretroviral drugs, the proportion of NNRTIs resistance is much higher than that of NRTIs and PIs in countries with NNRTIs-based first-line treatment, and if the percent of HIV-positive individuals harboring NNRTI resistance reaches above 10%, first-line treatments would need to be modified in these settings [13].

Guangxi in southwestern China, is one of the regions with the most severe HIV epidemic in China [14–17]. However, little comprehensive data are available on PDR. In this study, we conducted a large sample survey of HIV drug resistance to assess the level of PDR in recent years (2014–2020). HIV-positive individuals had genotyping and DRMs monitoring prior to initiation of antiretroviral therapy. A genetic transmission network was constructed to explore PDR related transmission.

## Methods

### Study design and study population

This was a cross-sectional study to estimate the prevalence of PDR in HIV-positive individuals that initiated ART in the Prefecture of Qinzhou, one of the regions with the most severe HIV epidemic in Guangxi. The study design was done according to the 2014 WHO protocol note for PDR [3]. Eligibility criteria were as follows: HIV-1 individuals newly diagnosed between January 1, 2014 and June 30, 2020, aged ≥ 18 years, signed the informed consent for PDR testing, and blood samples were successfully collected. Excluded from this study were persons who may have acquired drug resistance from previous antiretroviral drug exposure. After providing written informed consent, participants donated a single blood sample for HIV sequencing, HIV genotyping, and CD4+ T cell count assessment.

### Data collection

Basic sociodemographic data (age, sex, ethnic, education, marital status, and occupation), behavioral characteristics (route of HIV infection, condomless sexual behavior in the past 3 months), and baseline CD4+ cell count before ART were collected from the National HIV/AIDS Comprehensive Response Information Management System [18].

### Laboratory testing

Whole blood was sampled before the initiation of ART. For each person, CD4 cells were determined by the Alere PimaTM Analyzer (Abbott Laboratories, Germany). Frozen plasma (obtained by centrifuging whole blood) was isolated and sent on dry ice to the laboratory at National Center for AIDS/STD Control and Prevention (NCAIDS), Chinese Center for Disease Control and Prevention (CDC). RNA was extracted from 200 μl of plasma, amplified, and used to sequence the HIV pol region using an in-house Sanger sequencing protocol [8, 19]. The threshold for mixture base calling was 20%. DRMs screening was identified and interpreted according to the Stanford University Genotypic Resistance Interpretation (https://hivdb.stanford.edu/). Drug resistance to nucleoside reverse transcriptase inhibitors (NRTIs), non-nucleoside reverse transcriptase inhibitors (NNRTIs), and protease inhibitors (PIs) was defined as the detection of at least one antiretroviral virus drug in any drug class according to the WHO surveillance drug resistance guideline list [3]. Drug susceptibility was classified into four categories depending on mutation scores: susceptible—(< 15), low—(15–29), intermediate—(30–59), and high-level (≥ 60) resistance. Any strain with a mutation score ≥ 15 was considered to be PDR.

### Genetic network inference

The HIV genetic transmission network was inferred with HIV-TRACE [20], establishing putative transmission links between all sequences. We aligned HIV pol sequences to an HXB2 reference sequence (coordinates: 2253–3869) and calculated the pairwise genetic distances under the Tamura-Nei 93 (TN93) model [21]. Genetic networks with different genetic distances (range 0.1–1.5%) were established in order to find the most suitable genetic distance threshold that could identify the maximum number of clusters and links in the genetic network. However, Different genetic distance thresholds entertain different purposes. For example, a genetic distance of 0.5% would indicate that a strain would need a maximum time of 2–3 years in order to allow five different nucleotides to evolve, hence this threshold would be more aligned with rapid and more recent transmission events [22, 23]. The aim of the study was to analyze transmission relationships associated with recent and rapid transmission, so 0.5% was preferentially selected as the optimal genetic distance threshold. To avoid potential deviation caused by convergent evolution, the genetic transmission network was reestablished after codons associated with drug resistance mutations in PR and RT were removed based on the main drug resistance mutation of HIV-1 recently updated [20, 24], but the resulting network was unchanged. Molecular network was processed using the Cytoscape 3.5.2 software. Chord Diagram was created using the HIV-Trace that visualized trajectories of links between sequences in the molecular network.

### Statistical analysis

Statistical analyses were done in SAS V9.4 (SAS Institute Inc., Cary, NC, USA). Categorical variables are presented as number of cases and percentages, while continuous variables are expressed as the mean ± standard deviation (SD). Categorical variables which are presented as the number of cases and percentages were compared using the Pearson’s χ^2^ test or Fisher’s exact test, while continuous variables are expressed as the mean ± standard deviation (SD) and compared using the non-parametric Mann–Whitney U-test and Kruskal–Wallis test, where appropriate. Univariate and multivariate logistic regression models were used to explore associations between PDR with demographic and clinical variables applied to each subregion. Specifically, all the variables that were statistically significant in the univariate analysis were included in the multivariate logistic regression model, then select the variables independently associated with PDR step by step based on goodness-of-fit tests (e.g., AIC). *p* < 0.05 was considered statistically significant. The 95% CI of the overall PDR prevalence is estimated using the distribution of the sample rate (p) approximating a normal distribution. The calculation formula is:

$$\left(p-1.96{S}_{p},p+1.96{S}_{p}\right).$$

## Results

### General characteristics of the study population

Table 1 summarizes the baseline characteristics of 3262 eligible HIV-positive individuals that initiated ART in Qinzhou in the years 2014–2020. Most persons were over 50 years of age (55.1%, 1799/3262), male (73.4%, 2396/3262), Han (89.8%, 2930/3262), educated up to primary school level or below (56.2%, 1833/3262), married or cohabiting (59.8%, 1953/3262), and farmers (79.3%, 2588/3262). The self-reported risk factors for HIV-1 infection were mainly heterosexual intercourse (94.5%, 3082/3262), with 115 (3.5%) and 65 (2.0%) cases reported via injecting drug use or in men who have sex with men, respectively. A total of 2533 (77.7%) HIV-positive individuals had a CD4+ cell count < 350 cells/mm^3^ before ART and 669 persons (20.5%) had a CD4+ cell count ≥ 350 cells/mm^3^. The proportions of the subtypes CRF01_AE, CRF07_BC, CRF08_BC, CRF55_01B, B, and others were 56.7% (1849/3262), 10.2% (331/3262), 27.4% (895/3262), 0.9% (30/3262), 0.4% (12/3262), and 4.5% (145/3262), respectively.

**Table 1:** Characteristics of HIV-positive individuals in southwestern China, 2014–2020

Variable	Number	%
Total	3262	100
Age (years)		
18–29	305	9.4
30–49	1158	35.5
≥ 50	1799	55.1
Sex		
Female	866	26.6
Male	2396	73.4
Ethnicity		
Han	2930	89.8
Zhuang	290	8.9
Others	42	1.3
Education		
Primary and below	1833	56.2
Junior and high	1429	43.8
Marital status		
Single	668	20.5
Married or cohabiting	1953	59.8
Divorced or widowed	641	19.7
Occupation		
Farmer	2588	79.3
Others	674	20.7
Route of HIV infection		
Heterosexual intercourse	3082	94.5
Men who have sex with men	65	2.0
Injection drug use	115	3.5
Baseline CD4+ cell count before ART (cells/mm^3^)		
< 350	2533	77.7
≥ 350	669	20.5
Missing	60	1.8
HIV-1 genotype		
CRF01_AE	1849	56.7
CRF07_BC	331	10.2
CRF08_BC	895	27.4
CRF55_01B	30	0.9
B	12	0.4
Others	145	4.5

### Pretreatment HIV drug resistance

**Fig. 1:**
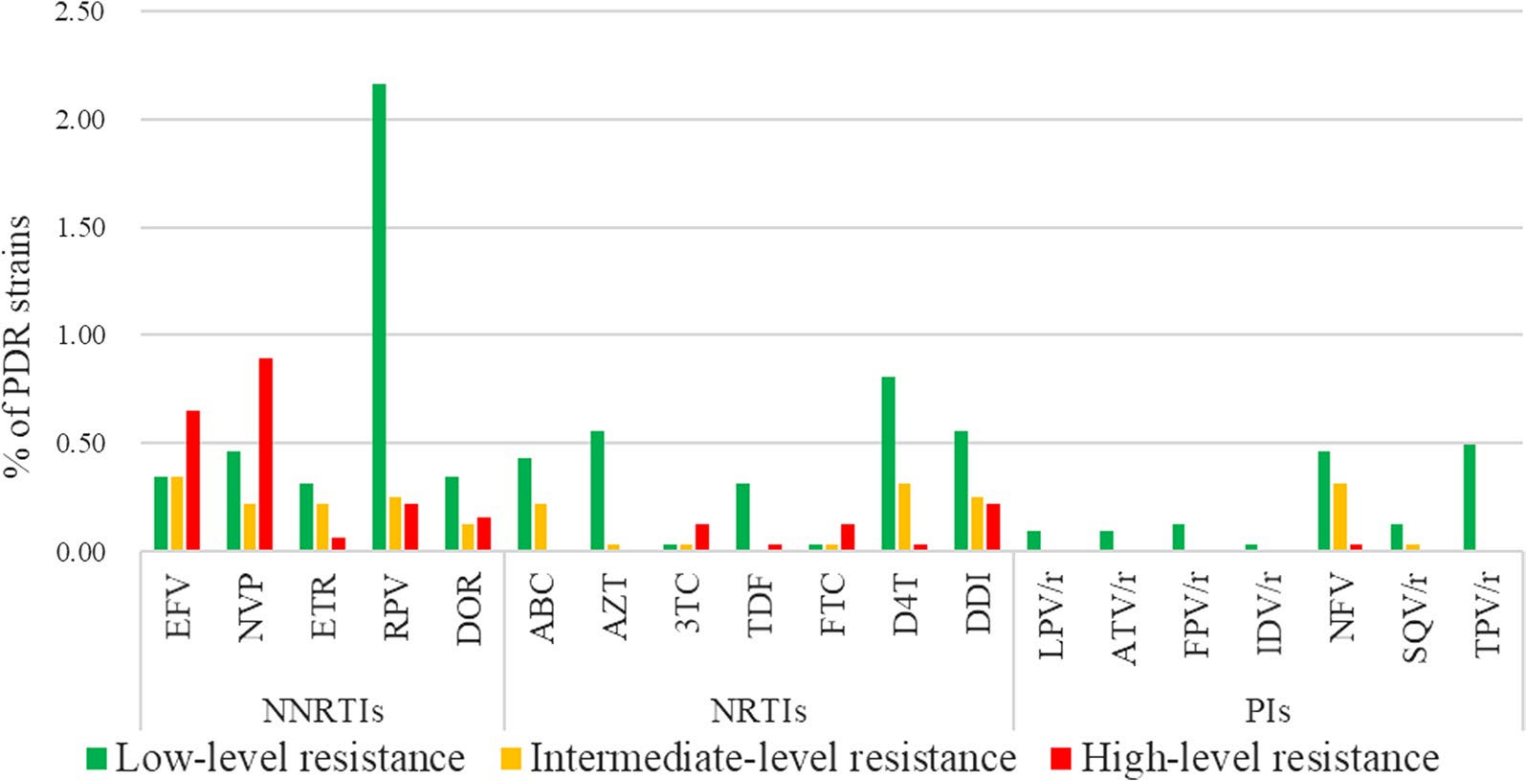
PDR (%) and levels of HIV-1 PDR to different antiretroviral drugs

The overall prevalence of PDR was 6.0% (194/3236, 95% CI 5.1–6.8%). The PDR frequency to NNRTI (3.3%, 106/3236) was much higher than that of NRTI (1.7%, p < 0.001) and PI (1.2%, p < 0.001). The most frequent NNRTI associated mutation was E138A/G/K, which was observed in 1.9% (60/3236) of HIV-positive individuals followed by K103N (0.4, 14/3236) and V179D/E (0.4%, 13/3236). K70E/T (0.3%, 9/3236) was the most prevalent NRTI associated mutation, followed by T215S/D/A (0.2%, 8/3236) and V75M/A (0.2%, 8/3236) (Table 2). Within NNRTI agents, PDR to RPV (2.6%, 85/3236) was more common than to NVP (1.6%, 51/3236) (χ^2^ = 8.68, *p* = 0.003) and EFV (1.3%, 43/3236) (χ^2^ = 14.06, *p* < 0.001), but the highest proportion of high-level resistance in resistant strains was NVP (56.9%, 29/51), followed by EFV (48.8%, 21/43). The proportion of PDR of ETR (0.6%, 19/3236) and DOR (0.6%, 20/3236) was similar (χ^2^ = 0.0258, *p* = 0.872) (Fig. 1). Within NRTI agents, PDR to 3TC and FTC were low at 0.1% (4/3236), yet these strains carried high-level resistance. Apart from that, low-level resistant strains were the main cases of ABC, AZT, TDF, D4T, and DDI, which were 0.4% (14/3236), 0.6% (18/3236), 0.3% (10/3236), 0.8% (26/3236), and 0.6% (18/3236), respectively. Within PI agents, the highest proportion of PDR was to NFV, with 15 (15/3236), 10 (10/3236), and 1(1/3236) in the low, middle and high-level resistance, respectively. There was no HIV-1 strain resistant to DRV/r found in our study (Fig. 1). PDR did not significantly increase from 2014 to 2020 (*p* = 0.317, Mantel–Haenszel χ^2^ linear trend test) (Additional file 1).

**Table 2:** Pretreatment HIV drug resistance in studied populations

Antiretroviral drug	Number	Percentage, % (95% CI)	HIV drug resistance mutations, N (%)
Total	194	6.0 (5.1–6.8)	
NNRTIs^a^	106	3.3 (2.6–3.9)	E138A/G/K, 60 (1.9) K103N, 14 (0.4) V179D/E, 13 (0.4) V108I, 8 (0.2) G190A/E, 7 (0.2) Y181C/I, 7 (0.2) V106I/M, 6 (0.2) A98G, 3 (0.1) Y188L/H, 3 (0.1)
EFV^d^^,e^	43	1.3 (0.9–1.7)	
NVP^d,^^e^	51	1.6 (1.1–2.0)	
ETR^e^	19	0.6 (0.3–0.8)	
RPV^e^	85	2.6 (2.1–3.2)	
DOR	20	0.6 (0.4–0.9)	
NRTIs^b^	54	1.7 (1.2–2.1)	K70E/T, 9 (0.3) T215S/D/A, 8 (0.2) V75M/A, 8 (0.2) T69GNS/D, 7 (0.3) L74I/V, 6 (0.2) M184V/I, 4 (0.1) L210W, 4 (0.1) M41L, 4 (0.1) D67N, 3 (0.1)
ABC^d^^,e^	21	0.7 (0.4–0.9)	
AZT^d^^,e^	19	0.6 (0.3–0.8)	
3TC^d^^,e^	6	0.2 (0.1–0.3)	
TDF^d^^,e^	11	0.3 (0.1–0.5)	
FTC^d^^,e^	6	0.2 (0.1-.03)	
D4T^d^^,e^	37	1.1 (0.8–1.5)	
DDI^d^	33	1.0 (0.7–1.4)	
PIs^c^	40	1.2 (0.9–1.6)	M46L/I/V, 20 (0.6) Q58E, 15 (0.5) G73S/CFV, 3 (0.1) L10F, 2 (0.1)
LPV/r^d^^,e^	3	0.1 (0–0.2)	
ATV/r^d^^,e^	3	0.1 (0–0.2)	
DRV/r^d^^,e^	0	0	
FPV/r	4	0.1 (0–0.2)	
IDV/r	5	0.2 (0–0.3)	
NFV/r	26	0.8 (0.5–1.1)	
SQV/r	5	0.2 (0–0.3)	
TPV/r	16	0.5 (0.3–0.7)	

^a^Non-nucleoside reverse transcriptase inhibitors

^b^Nucleoside/nucleotide reverse transcriptase inhibitors

^c^Protease inhibitors

^d^Antiretroviral drugs recommended by WHO

^e^Antiretroviral drugs available in China

### Factors associated with HIV PDR

Factors associated with HIV PDR are listed in Table 3. In a univariate logistic regression analysis, three factors were significantly associated with HIV PDR. Compared with persons aged ≥ 50, those aged 18–29 (OR: 2.29, 95% CI 1.46–3.60) or 30–49 (OR: 1.91, 95% CI 1.40–2.62) had significantly higher PDR rates. Unmarried status was also associated with PDR (OR: 2.00, 95% CI 1.24–3.22) compared with divorced or widowed. Persons harboring CRF08_BC compared with CRF07_BC were associated with an increased PDR rate (OR: 3.23, 95% CI 1.70–6.49). Furthermore, a multivariate logistic regression model showed that aOR for HIV-positive individuals aged 30–49 and 18–29 versus ≥ 50 were 1.79 (95% CI 1.28–2.50) and 2.82 (95% CI 1.73–4.82), respectively. CRF08_BC was also an important factor with an aOR of 3.23 (95% CI 1.58–6.59).

**Table 3:** Factors associated with pretreatment HIV drug resistance among HIV-positive individuals

Variable	Number	PDR, N (%)	*OR* (95% *CI*)	*p*	Adjusted *OR* (95% *CI*)	*p*
Total	3262	194 (6.0)				
Age (years)						
≥ 50	1799	76 (4.2)	1.00		1.00	
30–49	1158	90 (7.8)	1.91 (1.40–2.62)	< 0.001	1.79 (1.28–2.50)	< 0.001
18–29	305	28 (9.2)	2.29 (1.46–3.60)	< 0.001	2.82 (1.73–4.82)	< 0.001
Sex						
Male	2396	140 (5.8)	1.00			
Female	866	54 (6.2)	1.07 (0.78–1.48)	0.675		
Ethnic						
Zhuang	290	11 (3.8)				
Han	2930	181 (6.2)	1.67 (0.90–3.12)	0.106		
Others	42	2 (4.8)	1.27 (0.27–5.93)	0.763		
Education						
Primary or below	1833	99 (5.4)	1.00			
Junior or above	1429	95 (6.7)	1.25 (0.93–1.67)	0.136		
Marital status						
Divorced or widowed	641	27 (4.2)	1.00			
Married or cohabiting	1953	113 (5.8)	1.40 (0.91–2.15)	0.128		
Single	668	54 (8.1)	2.00 (1.24–3.22)	0.004		
Occupation						
Farmer	2588	153 (5.9)	1.00			
Others	674	41 (6.1)	1.03 (0.72–1.47)	0.866		
Route of infection						
Heterosexual intercourse	3082	180 (5.8)	1.00			
Men who have sex with men	65	3 (4.6)	0.78 (0.24–2.51)	0.677		
Injection drug use	115	11 (9.6)	1.71 (0.90–3.23)	0.102		
Baseline CD4+ cell count before ART (cells/mm^3^)						
≥ 350	669	34 (5.1)	1.00			
< 350	2533	157 (6.2)	1.23 (0.84–1.81)	0.279		
Missing	60	3 (5.0)	0.98 (0.29–3.30)	0.978		
HIV-1 genotype						
CRF07_BC	331	10 (3.0)	1.00		1.00	
CRF01_AE	1849	92 (5.0)	1.68 (0.87–3.26)	0.125	1.56 (0.77–3.17)	0.218
CRF08_BC	895	84 (9.4)	3.32 (1.70–6.49)	< 0.001	3.23 (1.58–6.59)	0.001
CRF55_01B	30	3 (10.0)	3.56 (0.93–13.74)	0.064	2.51 (0.60–10.44)	0.205
Others	145	5 (3.5)	1.15 (0.39–3.42)	0.806	1.03 (0.32–3.26)	0.964
B	12	0 (0.0)	–	–	–	–
Condomless sexual behavior in the past 3 months						
Yes	737	44 (6.0)	1.00			
No	2525	150 (6.0)	1.00 (0.70–1.41)	0.976		

– Not applicable

### Genetic transmission networks

**Fig. 2:**
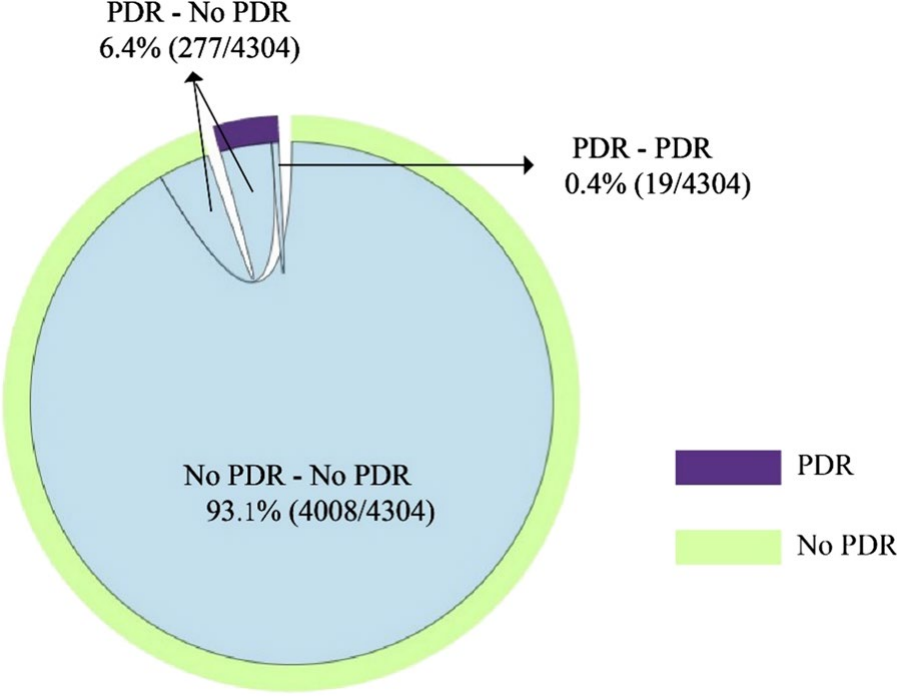
The composition of the links in the molecular transmission networks. (PDR–PDR: persons had PDR connect to persons had PDR in the molecular network; PDR–No PDR: persons had PDR connect to persons without PDR; No PDR–No PDR: persons without PDR connect to persons without PDR.)

**Fig. 3:**
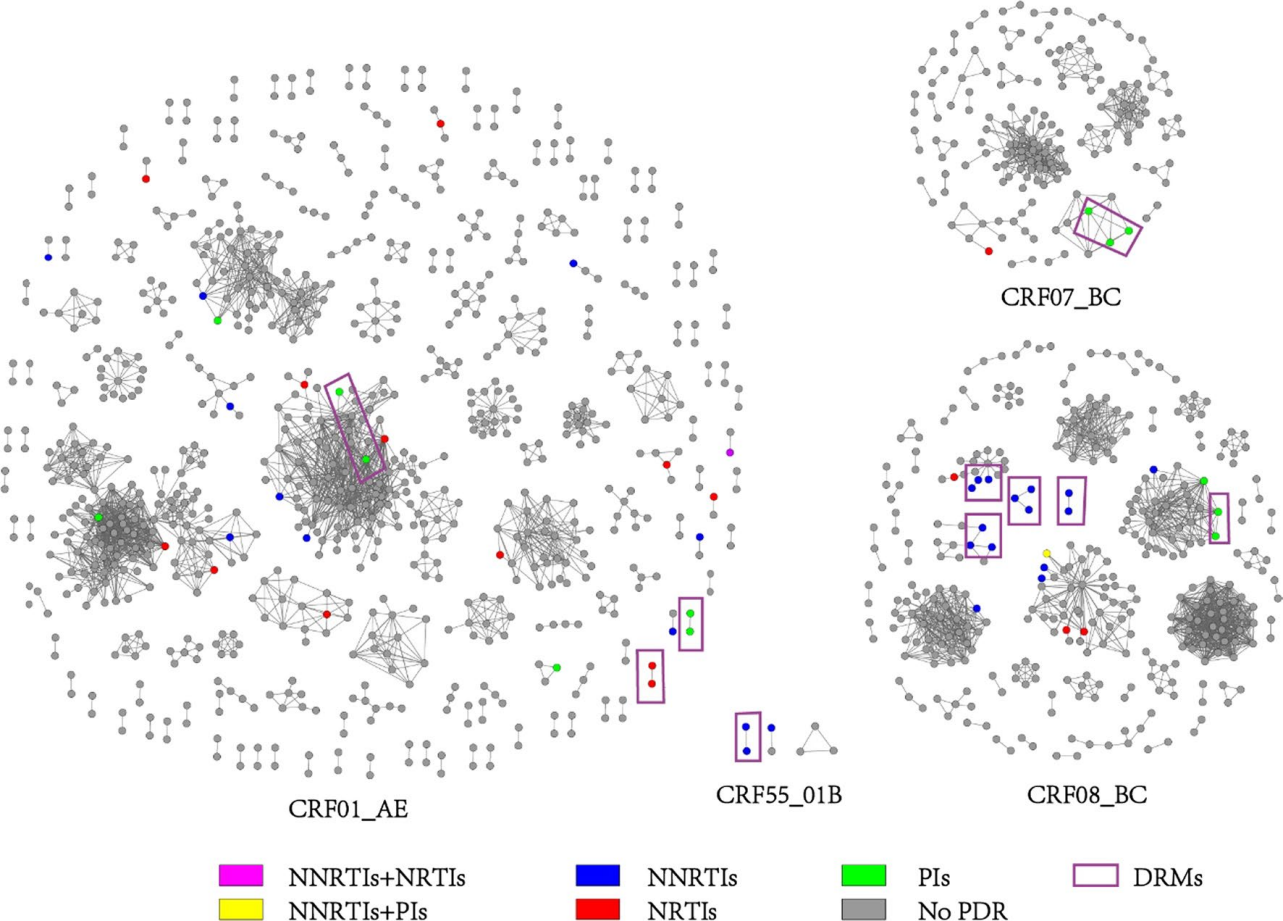
Pretreatment HIV drug resistance related molecular transmission networks. (Colored nodes represent PDR, and different colors represent different categories of drugs.)

Genetic networks with different genetic thresholds (range 0.1–1.5%) were established, it is found that the molecular network with 0.5% gene threshold had a high number of clusters to get a high-resolution molecular network (Additional file 2). With the most suitable threshold genetic distance of 0.5%, a total of 1429 (43.8%) sequences were linked forming molecular transmission clusters ranging in size from 2 to 119 individuals (257 dyads, 99 clusters with 3 or more individuals). Fifty-nine sequences with PDR were found in 29 clusters (10 dyads, 19 clusters of three or more individuals), mostly to NNRTIs (17 of 29 clusters). Of note, the percentage of individuals with PDR belonging to a cluster was lower than of those without PDR (30.4% versus 44.7%, p < 0.001) (Additional file 3); nevertheless, the links between individuals with PDR versus without were no different within clustering individuals (2.00 versus 2.00, p = 0.345) (Additional file 4). There was a total of 4304 links in the molecular network, of which, with 19 (0.4%) links were PDR connected to PDR, 277 (6.4%) were PDR linked to No-PDR, and 4008 (93.1%) were No-PDR connected to No-PDR, the latter of which accounted for the largest proportion (Fig. 2). However, same DRMs yielded 10 transmission networks, distributed among CRF01_AE (10.3%, 3/29), CRF07_BC (3.4%, 1/29), CRF08_BC (17.2%, 5/29), and CRF55_01B (3.4%, 1/29) (Fig. 3). In the CRF01_AE subtype cluster, 29 cases of PDR individuals were enrolled, distributed among 18 clusters, and 12, 9, and 7 were resistant to NRTIs, NNRTIs, and PIs, respectively. In addition, there was one case of NNRTIs and NRTIs resistance with mutation sites V106I, V179D, and V75M. For the CRF07_BC subtype cluster, four cases of PDR individuals were found in two clusters. They currently reside in the same county. Among them, three cases in the same cluster had a history of non-marital heterosexual contact, and all carried Q58E resistant mutation sites. In the CRF08_BC subtype cluster, 22 cases of drug resistance entered the networks, distributed in seven clusters, mainly NNRTIs resistance (68.2%, 15/22) with the E138A mutation site. One case harbored both NRTI and NNRTI mutations, and the mutations sites were K103N and M46I. Three PDR persons in the CRF55_01B subtype belonged to the same network, with E138G and V179E mutations (Fig. 3).

## Discussion

This cross-sectional study examined the prevalence of DRMs among 3236 ART-naive HIV-positive individuals in Qinzhou and obtained an overall PDR prevalence of 6.0% (194/3236, 95% CI 5.1–6.8%). According to the WHO definition of the prevalence of HIVDR, it is classified as low (< 5%), moderate (5–15%) and high (> 15%) [13]. The survey showed moderate (5–15%) rates of PDR in the Qinzhou region. It was higher than the prevalence in Qinzhou in 2012–2013 (2.6%, 1/38), as well as in other regions of Guangxi [25]. From 2014 to 2020, the overall prevalence of PDR had no downward trend in Qinzhou, Guangxi. Similarly, PDR rates are on the rise in the provinces with the most severe HIV epidemic such as Guangxi in China, similar to Dehong of Yunnan (3.48 to 9.48%) [26] and Liangshan of Sichuan (4.1 to 12.2%) [27, 28] from 2009 to 2017. In addition, the most common mutations of NNRTIs were E138A, K103N, and V179D mutations in this study, which was consistent with the study in a nationwide pilot survey of people with HIV/AIDS not receiving ART [28]. The most common mutation of NRTIs is the K70K mutation, which is the same as a previous study [29]. The recommended first-line regimen was AZT or D4T + 3TC + NVP in China [30]. Since 2010, D4T has been gradually replaced by AZT or TDF. Currently, the first-line therapy is TDF or AZT + 3TC + EFV or NVP, which has effectively reduced acquired drug resistance. However, with the exception of D4T, the prevalence of NNRTI PDR was 3.3% (95% CI 2.6–3.9) in this study, which is below the 10% threshold for changing the recommended first-line antiretroviral therapy [13]. Similarly, some studies in the past have shown that PDR is primarily driven by resistance to NNRTI, with higher resistance to EFV, NVP, and/or RPV in particular [31]. These findings suggest that the current available first-line ART regimens containing D4T, EFV, and/or NVP and/or RPV need to be revised. In addition, it is recommended that there be drug resistance testing and viral load measurements prior to ART initiation.

PDR may be influenced by many complex factors. This exploratory study found that age is an influential factor for PDR. Compared with people aged 50 and above, young people aged 18–29 are more likely to have pretreatment drug resistance, in line with studies in Jiangsu Province, Shandong Province, Guangxi, and Vietnam [32, 33]. Adherence to ART is likely lower in younger individuals, which increases their risk of harboring PDR. If these individuals engage in sexual activity, they could further transmit PDR strains to their sexual partners. However, whether it is due to increase sexual activity within certain individuals remains speculative and would require further study. Additionally, the frequency of mutations in the subtype CRF08_BC was significantly higher than that of CRF01_AE and CRF07_BC, which was consistent with the findings in Guangxi [11] and in Yunnan [34]. It has been suggested that the subtype CRF08_BC strain is more prone to base mismatches at certain sites during replication, leading to higher mutation rates, and persons carrying the CRF08_BC virus with mutations E138G, M184I, Y181C, Y188C, L100I, and may be highly resistant to antiretroviral drugs including NVP, EFV, or 3TC [35]. In addition, men who have sex with men and injection drug use were not found to be significant predictors of PDR in this study. In real-world research, the sample size of men who had sex with men or persons who inject drugs was low in this study, which could have reduced statistical power to determine whether these key populations were at risk of PDR. Nevertheless, the size of effect was still large in persons who inject drugs, which could be explained by the lower adherence to ART often observed in this population [28, 36].

In this study, the overall proportion belonging to a genetic cluster was not associated with PDR, as was observed in 13 provinces or cities in China (include high and moderate prevalence regions) [28], Liangshan Prefecture in Sichuan [27] and Shijiazhuang in Hebei [37]. However, there were 10 clusters containing same DRMs in the genetic networks identified in this study, while the size of these clusters appeared to expand over calendar years. In addition, some of the newly diagnosed HIV-positive individuals included in this study were not recently infected. They had not been treated with drugs after infection, and it is likely that the non-resistant stains became dominant and drug-resistant strains in minority populations were unable to be detected [38, 39], so the association between overall clustering rates and PDR was weakened. This result suggested that PDR may be transmitted among general groups in the future, and that interventions in total HIV-positive individuals are necessary to prevent the spread of drug-resistant strains in the region.

Our study has limitations. The sample transmission categories were based on self-reported information, which could not be validated. In addition, Sanger sequencing can only detect minority drug-resistant strains at a 15–20% prevalence in HIV viral populations of HIV-positive individuals and PDR could have been underestimated in this study [40]. In the future, we hope that next-generation sequencing can be used to identify HIV drug resistant variants at frequencies as low as 0.4%. Finally, although we did have access to the LAg-Avidity EIA test to determine recent HIV infection, this test was not used for the entire study population. As a result, we are unable to provide information on the recency of infection. Fortunately, the factor is randomized, so it does not have an impact on the determination of PDR.

In summary, large-scale drug resistance surveillance was carried out on HIV-positive individuals that initiated ART in Qinzhou, which has provided insights into the PDR prevalence, influencing factors, and potential transmission relationships of drug-resistant strains in the region. These findings indicate that there is an urgent need for surveillance programs of HIVDR and routine drug resistance testing in the clinical management of HIV-positive individuals. For ART-naïve persons, the results of drug resistance monitoring could guide clinicians in determining more effective, first-line therapeutic regimens. Also, interventions to improve ART adherence and investigations including more specific information on potential transmission could help reduce or even block continued transmission of drug-resistant strains. For national institutions, large-scale studies can help develop national guidelines for ART.

## Conclusions

In conclusion, large-scale drug resistance surveillance showed that the overall prevalence of PDR is moderate in Qinzhou. Numerous cases of same DRMs among genetically linked individuals in networks further illustrate the importance of surveillance research in the development of future strategies for mitigating PDR.

## Supplementary Information

**Additional file 1.** Changes in pretreatment HIV drug resistance among HIV-positive individuals in southwestern China, 2014–2020.

**Additional file 2.** Number of genetic transmission clusters and links, as a function of the TN93 distance threshold. (The epidemiologically plausible range of thresholds between 0.45% and 0.75% substitutions is highlighted in gray.)

**Additional file 3.** Difference in proportion of HIV-positive individuals belonging to genetic transmission networks who did and did not harbor PDR.

**Additional file 4.** Links difference between HIV-positive individuals with and without PDR in the genetic transmission networks.

## Data Availability

The datasets are available from the corresponding author on reasonable request.

## Abbreviations

HIV: Human immunodeficiency virus
PDR: Pretreatment drug resistance
DRM: Drug resistance mutation
WHO: World Health Organization
ART: Antiretroviral therapy
NNRTI: Non-nucleoside reverse-transcriptase inhibitor
NRTI: Nucleoside reverse-transcriptase inhibitor
PI: Protease inhibitor
TDR: Transmitted drug resistance
EFV: Efavirenz
NVP: Nevirapine
RPV: Rilpivirine
FTC: Emtricitabine
3TC: Lamivudine
ABC: Abacavir
DDI: Didanosine
D4T: Stavudine
HIVDR: HIV drug resistance
WHO: World Health Organization
OR: Odds ratio
aOR: Adjusted odds ratio
CI: Confidence interval
