# Supplementary material for: Survey of pretreatment HIV drug resistance and the genetic transmission networks among HIV-positive individuals in southwestern China, 2014–2020
Source: BMC Infect Dis. 2021 Nov 12;21:1153. doi: 10.1186/s12879-021-06847-5 (PMC8590229; doi:10.1186/s12879-021-06847-5)
Supplement: Supplementary file 1 — Additional file 1. Changes in pretreatment HIV drug resistance among HIV-positive individuals in southwestern China, 2014–2020. [file 12879_2021_6847_MOESM1_ESM.docx]

**Additional file 1** Changes in pretreatment HIV drug resistance among HIV-infected individuals in southwestern China, 2014-2020

| Year of newly diagnosed HIV-infected individuals | Number | PDR, N (%) | M-H *χ²* | *P*^b^ | *OR* (95% *CI*) | *P*^c^ |
| --- | --- | --- | --- | --- | --- | --- |
| Total | 3262 | 194 (6.0) | 1.00 | 0.317 |  |  |
| 2014 | 289 | 17 (5.9) |  |  | 1.00 |  |
| 2015 | 526 | 27 (5.1) |  |  | 0.87 (0.46-1.62) | 0.651 |
| 2016 | 593 | 33 (5.6) |  |  | 0.94 (0.52-1.72) | 0.848 |
| 2017 | 486 | 28 (5.8) |  |  | 0.98 (0.53-1.82) | 0.945 |
| 2018 | 488 | 34 (7.0) |  |  | 1.20 (0.66-2.19) | 0.555 |
| 2019 | 653 | 39 (6.0) |  |  | 1.02 (0.57-1.83) | 0.957 |
| 2020^a^ | 227 | 16 (7.1) |  |  | 1.21 (0.60-2.46) | 0.591 |

^a^ The diagnosis period is from January 1, 2020 to June 30, 2020.

^b^ Data are presented as N (%). Mantel-Haenszel (M-H) χ²linear trend test for the trend of pretreatment HIV drug resistance among newly diagnosed HIV-infected individuals over time.

^c^ Univariate logistic regression model was used to explore associations between pretreatment HIV drug resistance with Year of newly diagnosed HIV-infected individuals.
