# Supplementary material for: Survey of pretreatment HIV drug resistance and the genetic transmission networks among HIV-positive individuals in southwestern China, 2014–2020
Source: BMC Infect Dis. 2021 Nov 12;21:1153. doi: 10.1186/s12879-021-06847-5 (PMC8590229; doi:10.1186/s12879-021-06847-5)
Supplement: Supplementary file 2 — Additional file 2. Number of genetic transmission clusters and links, as a function of the TN93 distance threshold. (The epidemiologically plausible range of thresholds between 0.45% and 0.75% substitutions is highlighted in gray.) [file 12879_2021_6847_MOESM2_ESM.docx]

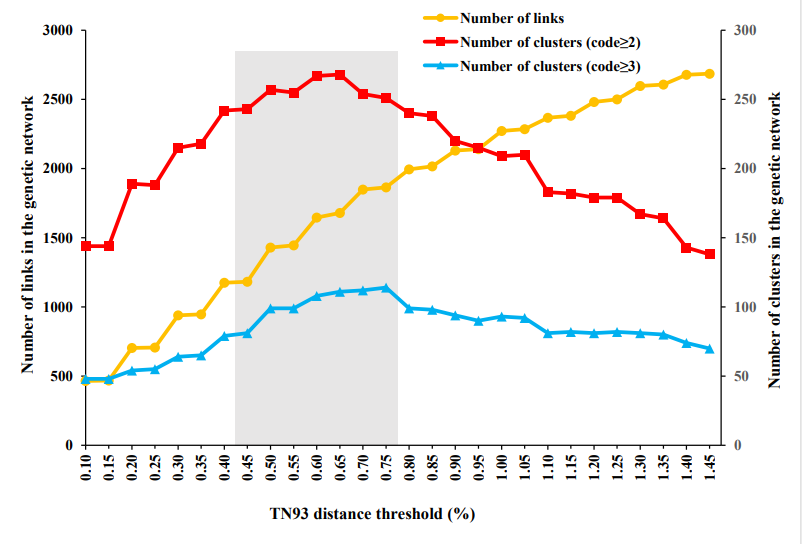


**Additional file 2** Number of genetic transmission clusters and links, as a function of the TN93 distance threshold. (The epidemiologically plausible range of thresholds between 0.45% and 0.75% substitutions is highlighted in gray.)
