# Supplementary material for: Survey of pretreatment HIV drug resistance and the genetic transmission networks among HIV-positive individuals in southwestern China, 2014–2020
Source: BMC Infect Dis. 2021 Nov 12;21:1153. doi: 10.1186/s12879-021-06847-5 (PMC8590229; doi:10.1186/s12879-021-06847-5)
Supplement: Supplementary file 3 — Additional file 3. Difference in proportion of HIV-positive individuals belonging to genetic transmission networks who did and did not harbor PDR. [file 12879_2021_6847_MOESM3_ESM.docx]

**Additional file 3** Difference in proportion of HIV-positive individuals belonging to genetic transmission networks who did and did not harbor PDR

|  | Number | Clustered, N | Percentage, % (*95% CI*) | *χ²* | *P* |
| --- | --- | --- | --- | --- | --- |
| Total | 3262 | 1429 |  | 15.03 | <0.001 |
| PDR | 194 | 59 | 30.4 (23.9-36.9) |  |  |
| No PDR | 3068 | 1370 | 44.7 (42.9-46.4) |  |  |
