# Supplementary material for: Survey of pretreatment HIV drug resistance and the genetic transmission networks among HIV-positive individuals in southwestern China, 2014–2020
Source: BMC Infect Dis. 2021 Nov 12;21:1153. doi: 10.1186/s12879-021-06847-5 (PMC8590229; doi:10.1186/s12879-021-06847-5)
Supplement: Supplementary file 4 — Additional file 4. Links difference between HIV-positive individuals with and without PDR in the genetic transmission networks. [file 12879_2021_6847_MOESM4_ESM.docx]

**Additional file 4** Links difference between HIV-infected individuals with and without PDR in the genetic transmission networks

|  | PDR (n=59) | No PDR (n=1370) | *Z* | *P* |
| --- | --- | --- | --- | --- |
| Link*, Median (IQR) | 2.00 (1, 6) | 2.00 (1, 7) | -0.94 | 0.345 |

* the number of links between sequences in the molecular network
